# Supplementary material for: Dynalign II: common secondary structure prediction for RNA homologs with domain insertions
Source: Nucleic Acids Res. 2014 Nov 21;42(22):13939–48. doi: 10.1093/nar/gku1172 (PMC4267632; doi:10.1093/nar/gku1172)
Supplement: SUPPLEMENTARY DATA [file supp_gku1172_nar-02021-z-2014-File012.zip › manual/RNA_class/html/classdesign.html]

RNAstructure Classes: design Class Reference

- Main Page
- Classes
- Files

- Class List
- Class Hierarchy
- Class Members

# design Class Reference

`#include <design.h>`

Inherits RNA.

List of all members.

|  |  |
| --- | --- |
|  |  |
| Public Member Functions | |
|  | design (const char filename[], const bool IsRNA=true) |
|  | Constructor. Programmer provides a filename for a ct file. |
| int | design\_sequence (const double pernucdefect, const bool random, const int maxdepth=5) |
| Private Member Functions | |
| void | decompose (int nucstart, int nucend, int currentdepth, int maxdepth, int numbering, int \*\*tree, int missingstart=0, int missingend=0) |
| bool | closeenoughtocut (int i, int j, int nucstart, int nucend, int missingstart, int missingend, double CLOSENESS) |
| void | marktree (int beststart, int bestend, int nucstart, int nucend, int missingstart, int missingend, int numbering, int currentdepth, int \*\*tree) |
| void | bestdecomposition (int nuctstart, int nucend, int currentstart, int currentend, int \*beststart, int \*bestend, int missingstart, int missingend) |
| void | SelectSequence (int \*\*tree, bool random, int depth, const double pernucdefect, long seed=1) |
| void | FindFragments (int \*\*tree, int level, int start, int stop, int missingstart, int missingstop, vector< int > \*stackstart, vector< int > \*stackend, vector< int > \*stackmissingstart, vector< int > \*stackmissingend, vector< int > \*stackfragmentdepth) |
| void | FillSequence (int start, int end, int missingstart, int missingend, bool random, randomnumber \*dice) |
| int | MapNuctoFragment (int j, int start, int missingstart, int missingend) |

---

## Constructor & Destructor Documentation

|  |  |  |  |  |
| --- | --- | --- | --- | --- |
| design::design | ( | const char | *filename*[], |  |
|  |  | const bool | *IsRNA* = `true` |  |
|  | ) |  |  |  |

Constructor. Programmer provides a filename for a ct file.

Read a ct with name filename. This is a template for the structure. The sequence in the ct file will be ignored. If IsRNA is true (default), this is RNA, and it is DNA otherwise.

**Parameters:**
:   |  |  |  |
    | --- | --- | --- |
    |  | *Filename* | is a null terminated cstring that provides the filename and path. |
    |  | *isRNA* | is a bool that is true for RNA (default value) and false for DNA. |

---

## Member Function Documentation

|  |  |  |  |  |
| --- | --- | --- | --- | --- |
| void design::bestdecomposition | ( | int | *nuctstart*, |  |
|  |  | int | *nucend*, |  |
|  |  | int | *currentstart*, |  |
|  |  | int | *currentend*, |  |
|  |  | int \* | *beststart*, |  |
|  |  | int \* | *bestend*, |  |
|  |  | int | *missingstart*, |  |
|  |  | int | *missingend* |  |
|  | ) |  |  | `[private]` |

|  |  |  |  |  |
| --- | --- | --- | --- | --- |
| bool design::closeenoughtocut | ( | int | *i*, |  |
|  |  | int | *j*, |  |
|  |  | int | *nucstart*, |  |
|  |  | int | *nucend*, |  |
|  |  | int | *missingstart*, |  |
|  |  | int | *missingend*, |  |
|  |  | double | *CLOSENESS* |  |
|  | ) |  |  | `[private]` |

|  |  |  |  |  |
| --- | --- | --- | --- | --- |
| void design::decompose | ( | int | *nucstart*, |  |
|  |  | int | *nucend*, |  |
|  |  | int | *currentdepth*, |  |
|  |  | int | *maxdepth*, |  |
|  |  | int | *numbering*, |  |
|  |  | int \*\* | *tree*, |  |
|  |  | int | *missingstart* = `0`, |  |
|  |  | int | *missingend* = `0` |  |
|  | ) |  |  | `[private]` |

|  |  |  |  |  |
| --- | --- | --- | --- | --- |
| int design::design\_sequence | ( | const double | *pernucdefect*, |  |
|  |  | const bool | *random*, |  |
|  |  | const int | *maxdepth* = `5` |  |
|  | ) |  |  |  |

Design the sequence. This function will choose a sequence will low ensemble defect to fold to the structure read in the constructor. The sequence is stored in the underlying RNA class.

**Parameters:**
:   |  |  |  |
    | --- | --- | --- |
    |  | *pernucdefect* | is the maximum allowed ensemble defect per nucleotide. |
    |  | *random* | determines the method for choosing sequence. If false, use pre-selected sequences, and otherwise (true), randomly choose the sequence. |
    |  | *maxdepth* | is the mximum extent to which the structure will be sub-divided in the binary decomposition. The default is 5. |

**Returns:**
:   An int error code that can be resolved to a message using RNA::GetErrorMessage. A return of 0 means no errors.

|  |  |  |  |  |
| --- | --- | --- | --- | --- |
| void design::FillSequence | ( | int | *start*, |  |
|  |  | int | *end*, |  |
|  |  | int | *missingstart*, |  |
|  |  | int | *missingend*, |  |
|  |  | bool | *random*, |  |
|  |  | randomnumber \* | *dice* |  |
|  | ) |  |  | `[private]` |

end else, (j!=missingstart)

|  |  |  |  |  |
| --- | --- | --- | --- | --- |
| void design::FindFragments | ( | int \*\* | *tree*, |  |
|  |  | int | *level*, |  |
|  |  | int | *start*, |  |
|  |  | int | *stop*, |  |
|  |  | int | *missingstart*, |  |
|  |  | int | *missingstop*, |  |
|  |  | vector< int > \* | *stackstart*, |  |
|  |  | vector< int > \* | *stackend*, |  |
|  |  | vector< int > \* | *stackmissingstart*, |  |
|  |  | vector< int > \* | *stackmissingend*, |  |
|  |  | vector< int > \* | *stackfragmentdepth* |  |
|  | ) |  |  | `[private]` |

|  |  |  |  |  |
| --- | --- | --- | --- | --- |
| int design::MapNuctoFragment | ( | int | *j*, |  |
|  |  | int | *start*, |  |
|  |  | int | *missingstart*, |  |
|  |  | int | *missingend* |  |
|  | ) |  |  | `[private]` |

|  |  |  |  |  |
| --- | --- | --- | --- | --- |
| void design::marktree | ( | int | *beststart*, |  |
|  |  | int | *bestend*, |  |
|  |  | int | *nucstart*, |  |
|  |  | int | *nucend*, |  |
|  |  | int | *missingstart*, |  |
|  |  | int | *missingend*, |  |
|  |  | int | *numbering*, |  |
|  |  | int | *currentdepth*, |  |
|  |  | int \*\* | *tree* |  |
|  | ) |  |  | `[private]` |

|  |  |  |  |  |
| --- | --- | --- | --- | --- |
| void design::SelectSequence | ( | int \*\* | *tree*, |  |
|  |  | bool | *random*, |  |
|  |  | int | *depth*, |  |
|  |  | const double | *pernucdefect*, |  |
|  |  | long | *seed* = `1` |  |
|  | ) |  |  | `[private]` |

end else, (j!=missingstart)

---

The documentation for this class was generated from the following files:

- E:/RNAstructure/RNA\_class/design.h- E:/RNAstructure/RNA\_class/design.cpp

---

Generated on Mon Apr 23 16:48:03 2012 for RNAstructure Classes by 
 1.5.7.1
